# A Splint-to-CT Data Registration Strategy for Maxillary Navigation Surgery

**DOI:** 10.1155/2020/8871148

**Published:** 2020-12-04

**Authors:** Shinsuke Yamamoto, Shigeo Hara, Toshihiko Takenobu

**Affiliations:** ^1^Department of Oral and Maxillofacial Surgery, Kobe City Medical Center General Hospital, Kobe, Hyogo 650-0047, Japan; ^2^Department of Pathology, Kobe City Medical Center General Hospital, Kobe, Hyogo 650-0047, Japan

## Abstract

Computer-assisted navigation plays an important role in modern craniomaxillofacial surgery. Although headpins and skull posts are widely used for the fixation of the reference frame, they require the use of invasive procedures. Headbands are easily displaced intraoperatively, thus reducing the accuracy of the surgical outcome. This study reported the utility of a novel splint integrated with a reference frame and registration markers for maxillary navigation surgery. A maxillary splint with a 10 cm resin handle was fabricated before surgery, to fix the reference frame to the splint. The splint was set after the incorporation of fiducial gutta-percha markers into both the splint and resin handle for marker-based pair-point registration. A computed tomography (CT) scan was acquired for preoperative CT-based planning. A marker-based pair-point registration procedure can be completed easily and noninvasively using this custom-made integrated splint, and maxillary navigation surgery can be performed with high accuracy. This method also provides maximum convenience for the surgeon, as the splint does not require reregistration, and can be removed temporarily when required. The splint-to-CT data registration strategy has potential applicability not only for maxillary surgery but also for otolaryngologic surgery, neurosurgery, and surgical repair after craniofacial trauma.

## 1. Introduction

Computer-assisted navigation has been used in a wide range of craniomaxillofacial surgeries, including facial deformity correction, facial trauma management, temporomandibular joint arthroplasty, tumor resection, removal of foreign bodies, and dental implantation [1–9]. Precise registration is important in navigation surgery, as it has direct repercussions on the precision of all subsequent navigation tasks [4, 5].

Several methods have been developed to improve registration accuracy during craniomaxillofacial surgery. The combination of headpins, skull posts, or headbands with positioning screws implanted into the maxillary alveolar bone has been recently used for marker-based registration [3, 4, 8]. However, the use of headpins and skull posts is invasive; headbands, on the other hand, can be easily displaced intraoperatively, thus reducing the accuracy of registration. The splint was integrated with the reference frame and registration markers to overcome the issues associated with maxillary navigation surgery. This system is noninvasive, and more accurate than that involving the use of a headband, since the reference frame can be mounted more rigidly and closer to the surgical field. The aim of the present case report was to evaluate the feasibility of a novel custom-made integrated splint for maxillary navigation surgery.

## 2. Case Report

A 70-year-old Japanese man with a medical history of fibrous dysplasia of the craniofacial bones was referred to the oral and maxillofacial surgery department of a general hospital in March 2019 with chief complaints of swelling and pain in the left buccal region. The patient provided informed consent prior to treatment commencement, and the study protocol was approved by the appropriate institutional ethics committee.

The patient had hypertension and was taking antihypertensive drugs. Clinical examination at the initial visit revealed facial asymmetry, mild swelling, and redness and tenderness over the left buccal region. No paresthesia was documented over the left buccal region. The left maxillary first molar was tender to percussion, and mild swelling and redness were observed on the buccal gingiva.

**Figure 1:**
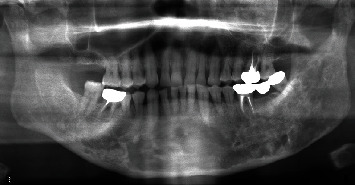
A panoramic radiograph obtained during the initial visit.

**Figure 2:**
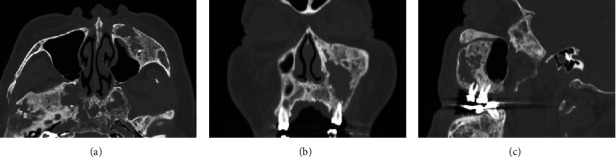
Computed tomography (CT) image obtained at the initial visit. CT shows bone hyperplasia and “ground-glass” appearance at the left maxilla, mandible, and sphenoidal bone, along with an osteolytic lesion extending from the root apex of the left maxillary first molar to the inferior aspect of the left infraorbital foramen and canal in the “ground-glass” lesion. Axial (a), coronal (b), and sagittal (c) CT views.

**Figure 3:**
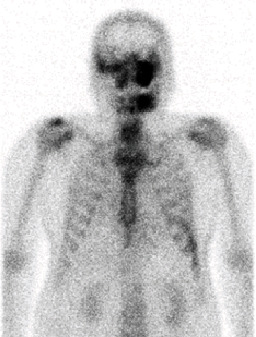
Bone scintigraphy findings obtained during the initial visit. Bone scintigraphy shows an accumulation of technetium medronic acid in the right skull base, cervical spine, left maxilla, and left mandible.

The panoramic radiograph revealed a “ground-glass” appearance in the left maxillary sinus and left mandibular body and ramus; a periapical radiolucency was observed with the left maxillary first premolar and first molar (Figure 1). Computed tomography (CT) revealed severe bone hyperplasia and “ground-glass” appearance with the left maxilla, mandible, and sphenoid bone, along with an osteolytic lesion extending from the root apex of the left maxillary first molar to the inferior aspect of the left infraorbital foramen and canal in the “ground-glass” lesion (Figure 2). Bone scintigraphy revealed the accumulation of technetium medronic acid in the right skull base, cervical spine, left maxilla, and left mandible (Figure 3).

The radiograph shows “ground-glass” appearance at the left maxillary sinus, mandibular body, and ramus. A periapical radiolucency is seen at the left maxillary first premolar and first molar.

**Figure 4:**
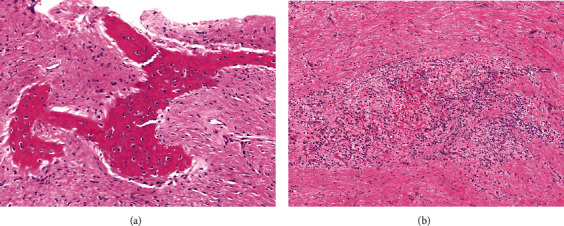
Surgical specimen. (a) A characteristic pattern of disconnected, bizarrely contoured dysplastic trabeculae enmeshed in primitive mesenchymal cells (hematoxylin and eosin, ×200). (b) Granulation tissue can be observed in the mesenchymal stroma (hematoxylin and eosin, ×100).

Ampicillin/sulbactam was prescribed for 7 days. A clinical diagnosis of left maxillary infection caused by a periapical lesion of the left maxillary first molar in a fibrous dysplasia lesion was established. The left maxillary first premolar and first molar were extracted, and the osteolytic lesion was curetted (while preserving the infraorbital neurovascular bundle) using a navigation system and novel navigation splint (described below) in October 2019. The patient declined correction for facial asymmetry via bone reduction. Fibrous dysplasia with granulation tissue was observed on pathological examination of the surgical specimen (Figure 4). Ampicillin/sulbactam was prescribed for 7 days postoperatively, and the patient was discharged from the hospital 4 days after surgery. The patient's 1-year postoperative course was uneventful.

## 3. Navigation Technique

**Figure 5:**
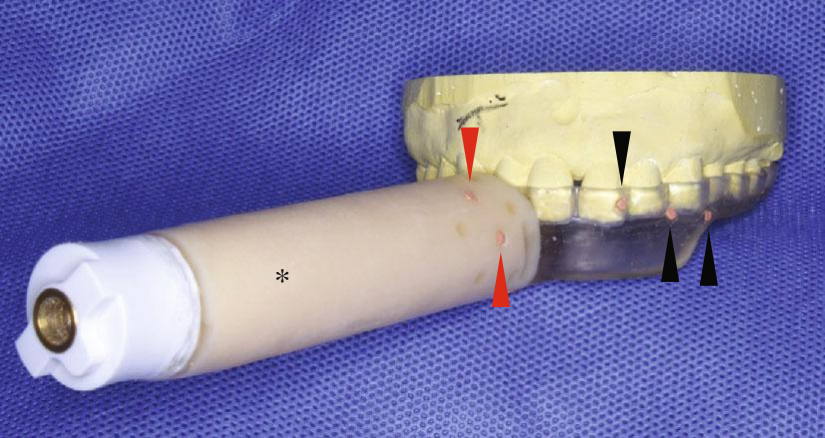
A custom-made splint. The 10 cm resin handle (with a connector), which was used to fix the reference frame (∗) and the gutta-percha markers are placed in asymmetric positions into both the splint (black arrowheads) and resin handle (red arrowheads) for marker-based pair-point registration.

**Figure 6:**
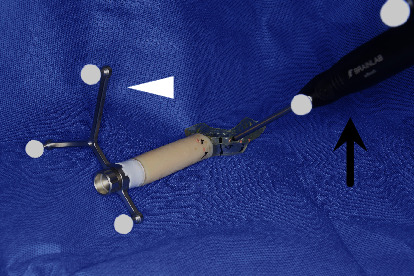
Marker-based pair-point registration using the custom-made splint, which is integrated with the reference frame and registration markers. The reference frame (white arrowhead) is fixed to the connector of the splint. The numbered landmarks (the 11 gutta-percha markers) are indicated within the optical tracking range of the navigation system and registered point-by-point in succession, using the pointer (black arrow).

**Figure 7:**
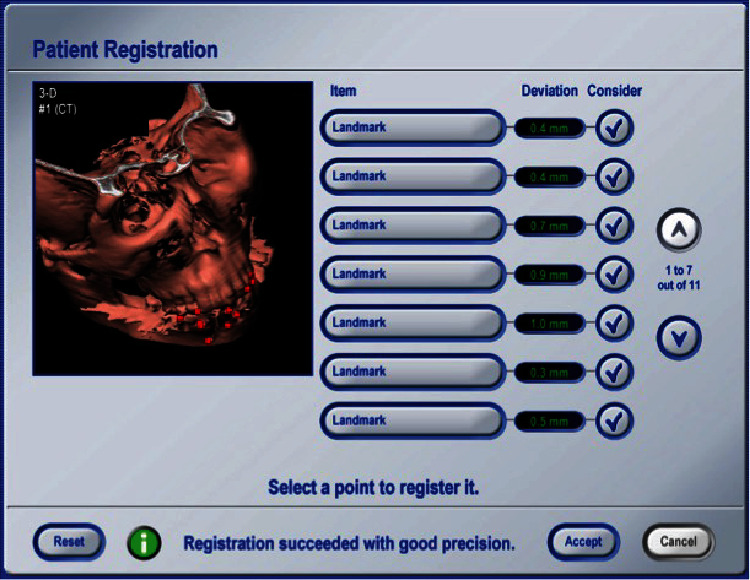
Screenshot of the navigation system during registration. The red points indicate the numbered landmarks (gutta-percha markers in the splint) for the marker-based pair-point registration. Deviations of each landmark are less than 1.0 mm.

A maxillary splint was fabricated, which extended from the right maxillary first molar to the left maxillary second molar, prior to surgery. The splint was composed of 3 mm thick Erkoloc-Pro plates (Erkodent, Pfalzgrafenweiler, Germany), and the resin extended to a point 1 cm below the incisal edges of the anterior teeth (Figure 5). A 10 cm resin handle with a connector, which was a reference star connector obtained from a reference headband (Brainlab AG, Feldkirchen, Germany), was used to fix the reference frame to the splint on the right side of the canine. The splint was set after the incorporation of 11 fiducial gutta-percha markers (each with a diameter of 1.5 mm) into the splint and resin handle for marker-based pair-point registration, and a CT scan was acquired [10]. Preoperative CT-based planning (iPlan CMF, Brainlab AG, Feldkirchen, Germany) entailed the determination of the region requiring curettage, as well as the registration and numbering of the 11 fiducial gutta-percha markers for the marker-based pair-point registration. The planning data were subsequently transferred to The Kick navigation system (Brainlab AG, Feldkirchen, Germany). The reference frame was fixed to the connector on the splint, and marker-based pair-point registration was performed, after the induction of general anesthesia and nasotracheal intubation. The practical procedure of the marker-based pair-point registration was as follows (splint-to-CT data registration): the numbered fiducial gutta-percha markers were indicated and registered point-by-point in succession within the optical tracking range of the navigation system, using the pointer (Figure 6). The splint was set on the maxillary dentition, and the presence of registration errors were determined with the pointer to assess the median positions of the maxillary central incisors and mesiobuccal line angles of the maxillary first molars on both sides. It took approximately 3 min to complete and confirm the marker-based pair-point registration before surgery; the mean fiducial registration error (FRE) was 0.68 ± 0.30 mm (Figure 7).

**Figure 8:**
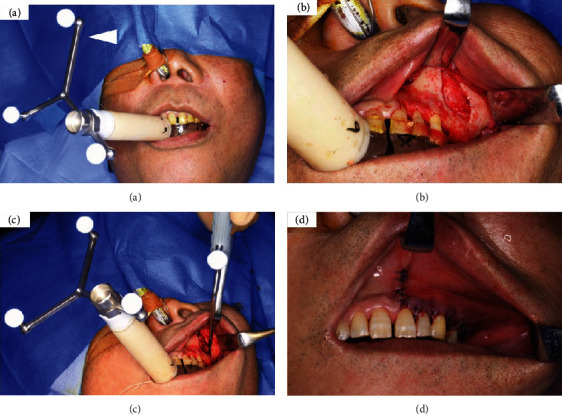
Intraoperative photographs. (a) The custom-made splint, which was integrated with the reference frame (white arrowhead), is set on the maxillary dentition. (b) The surgical field can be viewed clearly. (c) The positions of the osteolytic lesion are accurately identified in the surgical field using the pointer. (d) Placement of simple sutures at the vertical incision and horizontal mattress suture at the gingival margin.

**Figure 9:**
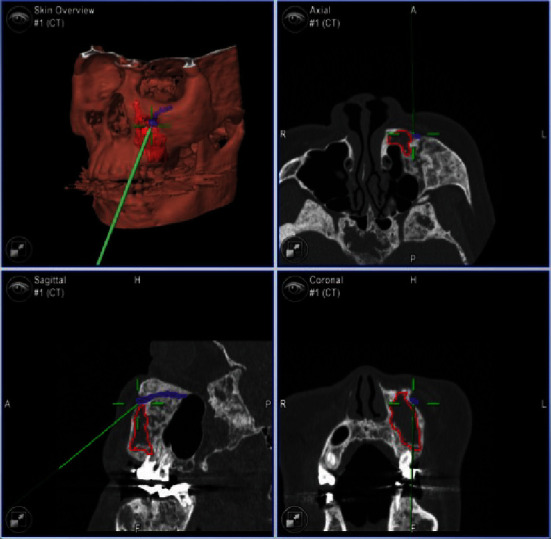
Intraoperative screenshot of the navigation system. The red line indicates the planned site for curettage. The contact of the pointer with the inferior border of the infraorbital foramen (purple line) is indicated.

The splint was temporarily removed to facilitate extraction of the left maxillary premolar and first molar. A three-sided flap was subsequently elevated, and the splint was reinserted (Figure 8). The positions of the infraorbital foramen and canal were accurately identified in the surgical field using the pointer, which facilitated the removal of the osteolytic lesion (Figure 9). Both the splint and reference frame were stable, as the splint was rigidly fixed to the maxillary dentition, which prevented the risk of a navigation system error. Simple sutures were placed at the vertical incision, and horizontal mattress sutures were placed at the gingival margin after the splint was removed (Figure 8).

## 4. Discussion

The findings derived from this case have two crucial implications. First, the marker-based pair-point registration procedure can be completed easily and noninvasively using a maxillary splint integrated with the reference frame and registration markers, and the use of such a splint allows maxillary navigation surgery to be performed with high accuracy.

Fibrous dysplasia is a common benign skeletal lesion that may involve one bone (monostotic) or multiple bones (polyostotic), which can occur throughout the skeleton with a predilection for the long bones, ribs, and craniofacial bones [11, 12]. However, most lesions can be treated with clinical observation and patient education [11, 12]. In the present case, an infection originated from an apical lesion and spread to the region affected by fibrous dysplasia, which necessitated curettage of the infected lesion and extraction of the causative teeth. Furthermore, a method facilitating safe removal of the lesions was needed, owing to the severe hypertrophy and deformity of the left maxillary bone caused by fibrous dysplasia, and the contact of the osteolytic lesion with the infraorbital foramen and canal.

Headbands, skull posts, or headpins are generally used in craniomaxillofacial navigation surgery [3, 4, 6, 8, 13]. The reference frame is first fixed to the patient's head with a headband, skull post, or headpin, which is followed by marker-free or marker-based registration. However, the use of headpins and skull posts is invasive, and a headband can easily be displaced intraoperatively. Furthermore, it is known that surgical precision decreases linearly with the distance from the reference markers [5, 7, 8]. The splint was integrated with the reference frame and registration markers to overcome the above-mentioned issues associated with maxillary navigation surgery. This system is noninvasive and more accurate than the use of a headband, since the reference frame can be mounted more rigidly and closer to the surgical field. Furthermore, as 11 fiducial gutta-percha markers were incorporated within the splint in asymmetric positions, marker-based pair-point registration could be performed in the patient's absence. The resin handle was also used for marker incorporation as the maxillary splint did not have adequate space to accommodate each of the 11 fiducial gutta-percha markers. The low mean FRE for splint registration (0.68 ± 0.30 mm) suggested that a widely spaced three-dimensional arrangement of the 11 fiducial gutta-percha markers contributed to the increase in the accuracy of the splint-to-CT data registration. This splint-to-CT data registration strategy is extremely precise and rapid; approximately 3 min were required to complete and confirm the marker-based pair-point registration before surgery, and there was no evidence of instability in the splint and reference frame that could have induced a navigation system error. It was also possible to preserve the infraorbital neurovascular bundle with this approach.

Second, this novel method provides maximum convenience for the surgeon, as the splint does not require reregistration, and can be temporarily removed when required. During craniomaxillofacial surgery, surgeons often change the patient's head position slightly to secure the surgical field; this is not possible when using a headpin. Any change in head positioning may result in the displacement of the headband, thus necessitating reregistration [14]. In contrast, the use of a splint allows changes in head positioning without displacing the reference frame; furthermore, the splint can be temporarily removed when it obstructs the surgical field, and reregistration is not required.

In the present case, the patient declined to undergo bone reduction for the correction of facial asymmetry caused by fibrous dysplasia. However, it would have been technically possible to perform this procedure by using the splint and navigation system to capture the mirror images of the opposite, unaffected side of the facial skeleton.

A limitation of oral splints is that their use is not feasible in patients who are edentulous or have few remaining teeth. Nevertheless, provided that a sufficient number of teeth are present to ensure the stable positioning of the splint, the results of this case report suggest that the splint-to-CT data registration strategy may be used not only in maxillary surgery, but also in otolaryngologic surgery, neurosurgery, and surgical repair after craniofacial trauma (especially zygomatic fracture and orbital floor fracture). Further studies with larger sample sizes are required to compare the effectiveness and reliability of this technique with conventional approaches.

## 5. Conclusion

A marker-based pair-point registration procedure can be completed easily and noninvasively, and maxillary navigation surgery can be performed with high accuracy by incorporating a custom-made integrated splint. This method also provides maximum convenience for the surgeon, as the splint does not require reregistration, and can be temporarily removed when required.
